# Altered activity in the nucleus raphe magnus underlies cortical hyperexcitability and facilitates trigeminal nociception in a rat model of medication overuse headache

**DOI:** 10.1186/s12868-019-0536-2

**Published:** 2019-10-21

**Authors:** Prangtip Potewiratnanond, Supang Maneesri le Grand, Anan Srikiatkhachorn, Weera Supronsinchai

**Affiliations:** 1 Interdisciplinary Program of Physiology, Graduate School, Chulalongkorn University, Bangkok, Thailand; 2 Department of Pathology, Faculty of Medicine, Chulalongkorn University, Bangkok, Thailand; 3 Faculty of Medicine, King Mongkut’s Institute of Technology Ladkrabang, Bangkok, Thailand; 4 Department of Physiology, Faculty of Dentistry, Chulalongkorn University, Bangkok, Thailand

**Keywords:** Acetaminophen, Cortical spreading depression, Extracellular recording, Nitroglycerin, Nucleus raphe magnus, Paracetamol, Trigeminal nucleus caudalis

## Abstract

**Background:**

The pathogenesis of medication overuse headache (MOH) involves hyperexcitability of cortical and trigeminal neurons. Derangement of the brainstem modulating system, especially raphe nuclei may contribute to this hyperexcitability. The present study aimed to investigate the involvement of the nucleus raphe magnus (NRM) in the development of cortical and trigeminal hyperexcitability in a rat model of MOH.

**Results:**

Chronic treatment with acetaminophen increased the frequency of cortical spreading depression (CSD) and the number of c-Fos-immunoreactive (Fos-IR) neurons in the trigeminal nucleus caudalis (TNC). In the control group, muscimol microinjected into the NRM increased significantly the frequency of CSD-evoked direct current shift and Fos-IR neurons in the TNC. This facilitating effect was not found in rats with chronic acetaminophen exposure. In a model of migraine induced by intravenous systemic infusion of nitroglycerin (NTG), rats with chronic exposure to acetaminophen exhibited significantly more frequent neuronal firing in the TNC and greater Fos-IR than those without the acetaminophen treatment. Muscimol microinjection increased neuronal firing in the TNC in control rats, but not in acetaminophen-treated rats. The number of Fos-IR cells in TNC was not changed significantly.

**Conclusion:**

Chronic exposure to acetaminophen alters the function of the NRM contributing to cortical hyperexcitability and facilitating trigeminal nociception.

## Background

Overconsumption of medication to abort headaches, either migraine specific agents, or narcotic or nonnarcotic analgesics, is a known factor for deteriorating headache symptoms in patients with primary headaches, especially migraine and tension-type headache. This overconsumption contributes to the development of a syndrome known clinically as “medication overuse headache” (MOH). MOH refers to a frequent headache (15 or more days/month) in a patient with pre-existing primary headaches and develops as a consequence of regular overuse (10 to 15 or more days/month, depending on medication) of acute or symptomatic headache medication for more than 3 months. It usually, but not invariably, resolves after overuse is stopped [1]. This condition is one of the most common chronic headaches with a prevalence of 1–2% worldwide [2].

The mechanism by which abortive medication worsens clinical headache remains unclear. One hypothesis is neuronal hyperexcitability [3]. Preclinical studies show that chronic analgesic drug exposure can increase neuronal excitability in the cerebral cortex and trigeminal nociceptive system. For instance, expanded cutaneous receptive fields and reduced thresholds to painful stimuli were demonstrated in rats with sustained morphine exposure [4]. Persistent administration of a triptan to rats elicited periorbital cutaneous allodynia [5]. An increase in frequency of cortical spreading depression (CSD) and number of CSD-evoked c-Fos-immunoreactive (Fos-IR) cells in the trigeminal nucleus caudalis (TNC) was demonstrated in rats receiving acetaminophen or dihydroergotamine [3, 6]. The hypothesis of cortical hyperexcitability is supported by clinical evidence. In patients with MOH, the amplitude of pain-related cortical potentials (PREP) evoked by electrical sensory stimulus on the forehead or a limb were greater than they were in a control group. The facilitation of both trigeminal and somatic PREP in those patients became normalized after drug withdrawal [7]. Patients with MOH also had larger amplitude somatosensory evoked potentials (SEP) than nonheadache control patients, and lacked SEP habituation [8].

Derangement of serotonergic systems in the raphe nuclei may contribute to this neuronal hyperexcitability. Preclinical studies showed that rats with depleted serotonin levels exhibited similar features to rats with chronic exposure to medication. These features include increased CSD susceptibility, facilitated trigeminal nociception, and upregulated pronociceptive messengers, such as nitric oxide [9, 10]. Some clinical observations indicate that the serotonin system is altered in patients with chronic headache both with and without medication overuse. For example, D’Andrea et al. [11] showed that the plasma level of tryptophan was significantly lower in patients with chronic migraine and chronic tension-type headache. Lower levels of platelet serotonin and upregulation of pronociceptive 5-HT_2A_ serotonin receptors was found in patients with MOH [12, 13].

The primary objective of the present study was to investigate the involvement of the nucleus raphe magnus (NRM) in the pathogenesis of MOH. We used two animal models of migraine, effected by KCl-evoked CSD and intravenous systemic nitroglycerin (NTG) infusion. Serotonergic function was inhibited by direct microinjection of the GABA_A_ agonist muscimol into the NRM. We measured trigeminal neuron activity by recording extracellular activity and counting the number of Fos-IR cells neurons in the TNC.

## Methods

### Animals

Adult male Wistar rats weighing 180–200 g were obtained from the National Laboratory Animal Center, Mahidol University. The rats were housed in cages with stainless-steel bases covered in wood shavings (4–5 animals per cage), at an ambient room temperature of 25–30 °C under a 12 h photoperiod. The rats were allowed unrestrained access to standard laboratory food and tap water. To limit the effect of nonspecific stress, all rats were acclimatized to daily handling for at least 3 days before experiments.

### Drugs and treatments

Acetaminophen (paracetamol) in solution (300 mg/2 mL) was purchased from T.P. Drug Laboratories (Bangkok, Thailand). The acetaminophen dose used in present experiments was 200 mg/kg as consistent with previous studies [3, 6, 7, 10]. Normal saline was administered to rats in a vehicle-treated control group. The volume of all drug injections was calculated according to standard criteria (intraperitoneally 10 mL/kg). Doses of acetaminophen were chosen based on the presence of efficacy without serious adverse effects. Intraperitoneal injections of the acetaminophen or saline vehicle control were administered at a similar time of day (8:00 to 9:00 a.m.). Muscimol was purchased from Calbiochem (Merck). Nitroglycerin was purchased from Pharmaland (Bangkok, Thailand). Each subgroup comprised 6–8 rats.

### Study design

Studies were divided into two experiments. The first was aimed at determining the modulating effect of NRM on CSD, CSD-induced cortical hyperemia, and CSD-evoked Fos-IR in the TNC. In this experiment, rats were divided into two groups, an acetaminophen-treated group and a vehicle-treated control group. Intraperitoneal injections were made on 30 consecutive days. On day 31, rats were prepared to record electrocortical activity and cortical blood flow. CSD was elicited by applying solid KCl to the exposed cortex. CSD and CSD-evoked changes in CBF recorded during the first 30 min after applying KCl were used as baseline data. Muscimol (10 nM, 0.2 µL) or 0.9% NaCl (0.2 µL), both of which contained Pontamine Sky Blue to mark the microinjection site, was microinjected into the NRM 30 min after eliciting a CSD, and electrocortical activity and cortical blood flow were recorded for a further 90 min. After completing the recordings, the brainstem was removed for immunohistochemistry to determine the expression of c-Fos.

A second experiment was designed to study the effect of inhibiting the NRM on NTG-evoked trigeminal nociception. Rats were divided into an acetaminophen-treated group and a vehicle-treated control group (n = 15 or 16 each respectively). The drug administration protocol was the same as for the CSD experiment. On day 31, rats were prepared for extracellular recording of neurons in the TNC. The spontaneous activity of neurons in the TNC was recorded for 10 min as a baseline and then NTG was infused intravenously (0.5 mg/kg, infusion rate 0.5 mL/h). One hour after the infusion, muscimol or 0.9% NaCl (n = 7 or 8 per subgroup) was microinjected into NRM. Activity of TNC neurons was continuously monitored for 2 h. In these two experiments, rats were randomly allocated into each treatment group using a computer-generated random table. All measurements were conducted by investigators who were blinded to the experimental treatments. All procedures were approved by the Ethics Committee of the Faculty of Dentistry, Chulalongkorn University (Animal use Protocol No. 1632001).

Rat livers were examined histologically with hematoxylin and eosin staining to exclude the influence of hepatotoxicity possibly induced by acetaminophen treatment. Indicators of hepatotoxicity were the presence of centrilobular or panacinar necrosis and sinusoidal congestion. Histology was conducted by investigators blinded to the rat treatments.

### Experiment 1

The effect of inhibiting the NRM on CSD, CSD-induced direct current shift, and CSD-evoked Fos-IR in the TNC.

#### Extracellular cortical activity recording and induction of CSD

Following a previously described protocol [24], 1 day after administering the final dose of acetaminophen, rats were anesthetized to a surgical plane with an intraperitoneal injection of sodium pentobarbital (60 mg/kg) such that the paw pinch withdrawal reflex was abolished. Their heads were fixed to a holder in a stereotaxic frame. For direct current recordings, we opened a small window in the left frontal bone of the cranium (2 mm wide, 3 mm anterior to bregma, and 2 mm lateral from the midline). The dura mater was then carefully reflected and a glass microelectrode to detect negative direct current potential was inserted into the frontal cortex to a depth of 1 mm using a hydraulic micromanipulator (Narishige, Tokyo, Japan). An Ag/AgCl reference electrode was placed on shaved skin on the back of the rat. Then a parietal window (7 mm posterior and 3 mm lateral to the bregma) was prepared. The dura was carefully opened using a 27-gauge needle under a surgical microscope. Solid KCl (3 mg) was applied to the cortex under the posterior craniotomy to initiate CSD. An analog electrical signal obtained was amplified (IX2-700, Dagan, Minneapolis, MN, USA) and converted to a digital form using a data acquisition system (PowerLab 4/35, ADInstruments, Bella Vista, New South Wales, Australia). Recordings were analyzed using matching software (LabChart 8, ADInstruments).

#### Microinjection procedure

To allow access to the NRM, a rectangular midline craniotomy was made over the cerebellum by careful grinding with a dental burr. Next, a microinjection needle was inserted into the NRM. The coordinates used for the NRM were − 11.6 mm anterior/posterior, 0 mm lateral, and − 10.5 mm ventral to bregma [25]. Muscimol (10 nM in 0.2 µL) or saline (0.2 µL) containing Pontamine Sky Blue was injected using a 5 µL Hamilton syringe. Injections were controlled at a constant rate over 1 min using a microsyringe pump controller (World Precision Instruments, Sarasota, FL, USA).

**Fig. 1:**
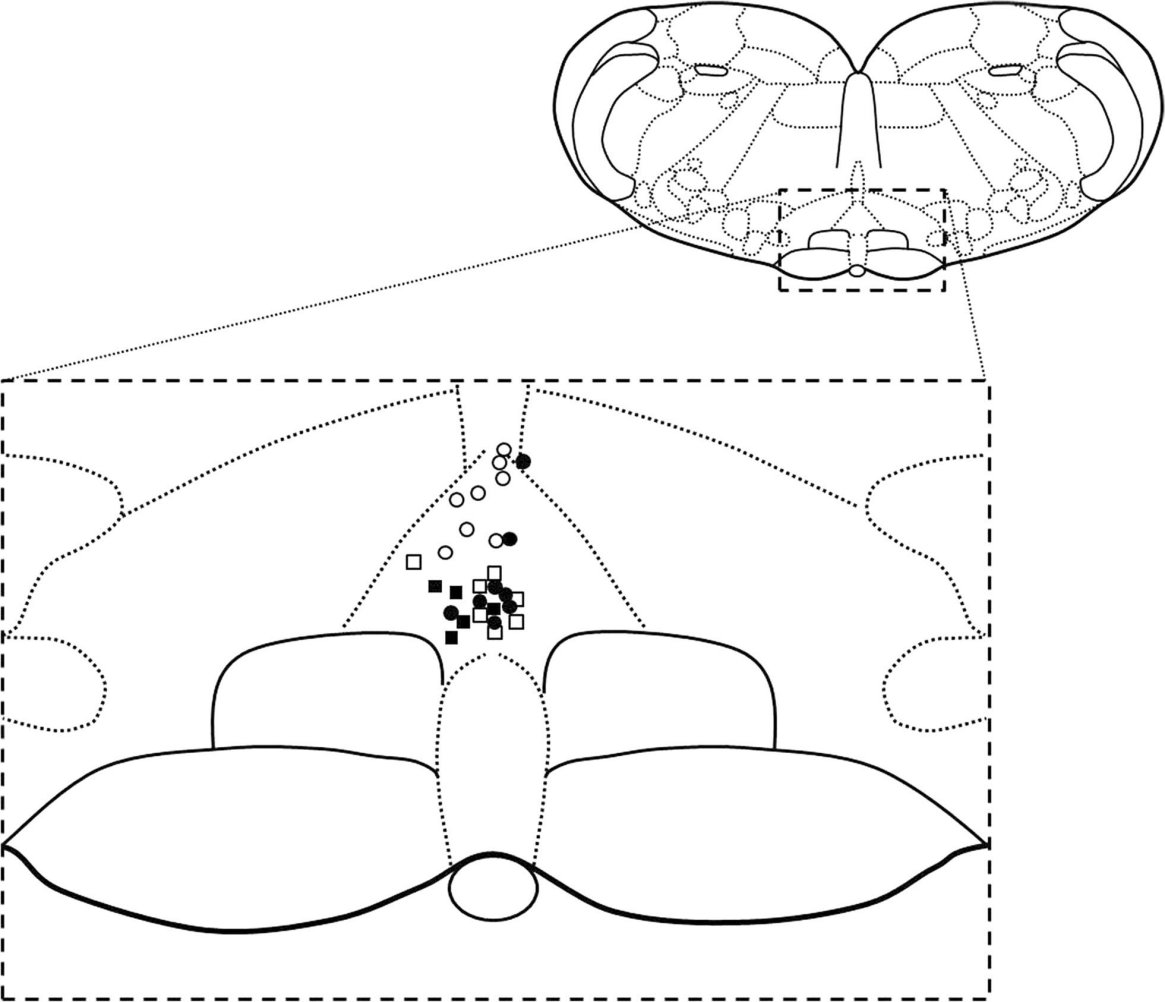
Schematic of a coronal brainstem section of a rat showing the sites of microinjection into the nucleus raphe magnus. Open circles represent microinjection sites of saline in the saline-treated vehicle-control rats; solid circles represent sites of saline microinjection in the chronic acetaminophen-treated rats; open squares represent sites of muscimol microinjection in the saline-treated vehicle-control rats; and solid squares represent sites of muscimol microinjection in the chronic acetaminophen-treated rats (The schematic is adapted from a Figure by (in Ref. [25]))

The microinjected-site within the NRM was assessed by checking histological sections after completing the experiment. Figure 1 shows the sites of microinjections in all groups. Data obtained from rats with microinjection sites outside of the NRM were excluded.

### Experiment 2

The effect of inhibiting the NRM on TNC neuronal activity and Fos-IR after being evoked by intravenous infusion of NTG.

#### Extracellular recording at the TNC

Rats were anesthetized to a surgical plane of anesthesia as before, and a tracheostomy performed. Their femoral artery and vein were cannulated to record blood pressure and administer drugs, respectively. Subsequently, the head of the rat was fixed in a stereotaxic frame. A midsagittal incision was made in the skin overlying the C1–C2 spinal cord. The laminar process of C1 was removed by laminectomy. The medullary brainstem was exposed by carefully cutting the overlying dura mater. Recording sites were located in an area 2.0–4.5 mm caudal to the obex, 0.8–2.5 mm lateral to the midline, and 0.2–1.0 mm from the surface of the brainstem. A glass recording electrode filled with 4 M NaCl was advanced using a hydraulic micromanipulator into the left side of the TNC with reference to an atlas of the rat brain [25]. Then, neurons were tested for convergent input from periorbital skin at the ipsilateral side by touching the skin with a cotton pledget. Neurons were classified by their response to electrical stimulation (0.5 Hz, 0.1 ms, 15–20 V) at the middle meningeal artery. A latency of 5–15 ms was used to indicate that received afferent input was most likely from Aδ-fibers (> 2 m/s) or C-fibers (0.5–2 m/s).

The signals obtained were amplified, bandpass filtered (DP-311, Warner Instruments, Hamden, CT, USA), and saved for offline analysis using LabChart 8 software. A Hum Bug Noise Eliminator was used (Quest Scientific, North Vancouver, BC, Canada). After identifying neurons in the TNC, spontaneous activity of the neurons was recorded for 10 min as a baseline. NTG was infused and TNC neuronal activity was continuously monitored. One hour after NTG infusion, muscimol or saline (0.2 µL) was microinjected into NRM. TNC activity was recorded for a further 1 h. Extracellular signaling in 20 min intervals was counted and reported as the percent change from the baseline value of the spontaneous neuronal activity recorded in the first 10 min as described above.

#### Nitroglycerin infusion

Nitroglycerin (0.5 mg/kg, 1 mL) was infused systemically at a constant rate of 0.5 mL/h into the femoral vein for 2 h by using syringe pump controller (Harvard Apparatus, Holliston, MA, USA). One hour after infusing NTG, muscimol or 0.9% NaCl was injected into NRM.

#### Immunohistochemistry

After completing the recording of CSD or TNC neuronal activity, rats were prepared for immunohistochemistry. The rats were killed humanely with an overdose of sodium pentobarbital (Nembutal, Ceva Animal Health, Bangkok, Thailand; 90–120 mg/kg intraperitoneally) and transcardial perfusion with phosphate buffer (250 mL) followed by 4% paraformaldehyde (250 mL). The occipital bone and laminar processes of C1–C2 were resected. The brainstems were carefully excised using a sharp scalpel blade and immediately immersed in 4% paraformaldehyde for 24 h to fix the tissue. Before preparing slices, the brainstems were stored in 30% sucrose solution in a phosphate buffer for 2 days. Multiple 30 µm thick transverse cross-sections from the brainstem (2–6 mm caudally from the tip of obex) were cut using a cryostat microtome (Leica, Nussloch, Germany) and separated into two segments, including 2 mm caudal from obex to C1 spinal nerve and C1 to C2 spinal nerve. Every third section from each segment was collected and processed for c-Fos immunocytochemistry. To avoid the bias related to the interpretation of three-dimensional information based on two-dimensional histomorphometry, the sections were collected systematically.

The sections were rinsed in three changes of phosphate-buffered saline (PBS) to remove embedding media. Next, sections were incubated with 50% ethanol for 30 min and 3% hydrogen peroxide in 50% ethanol for 30 min to quench endogenous peroxidase. After repeated rinses in PBS, nonspecific binding of the antibody was blocked by incubating tissues with 3% normal horse serum (Invitrogen, New Zealand) in PBS for 60 min at room temperature. After washing with PBS three times, the sections were incubated in a solution of c-Fos specific antibody (1:1000; rabbit polyclonal c-Fos antibody, Santa Cruz, Dallas, TX, USA) at 4 °C. After incubating overnight, the sections were rinsed three times with PBS. Then, the sections were incubated with labeled polymer-horseradish peroxidase anti-rabbit antibodies (Envision system, Dako, USA) for 30 min, followed by rinsing with PBS. Subsequently, all sections were incubated in substrate containing 3,3′-diaminobenzidine (DAB) (1 drop of DAB: 1 mL of substrate) for 10 min. The reaction was stopped by washing with PBS. Finally, these sections were mounted on glass slides, air-dried and covered with coverslips. We counted 20 sections per animal for Fos-IR cells in the Rexed lamina I–II. Only cell profiles with a visible staining on the focal plane were analyzed. Immunoreactive cells were defined as those with a dark-brown stain in their nucleus.

### Statistical analyses

All data are presented as mean ± standard deviation (SD). Statistical analyses were performed using a Mann–Whitney *U* test and Wilcoxon rank sum test. ANOVA for repeated measures was used for multiple comparisons. *p* < 0.05 was considered significant in tests of statistical inference. IBM SPSS Statistics for Windows (version 22, IBM Corp, Armonk, NY, USA) was used to analyze all data.

## Results

The general condition of the rats, such as body weight and food intake, was not altered by chronic administration of acetaminophen. Average body weight of rats at the end of experiments was 359.4 ± 23.1 g in the saline vehicle-treated control group and 346.7 ± 16.1 g in the acetaminophen-treated group. There was no significant difference between the average body weight of rats in any group. Histology demonstrated no hepatocellular necrosis, excluding liver toxicity. Infusion of NTG caused a minimal, less than 10%, drop in arterial blood pressure. There was no significant difference in average blood pressure between rats in any group in the present study. The same rats were assessed for both electrophysiological recording and immunohistochemical staining.

### Effect of inhibiting serotonergic systems in the NRM on CSD development

The number of waves of CSD during a 30 min baseline period was increased significantly in rats chronically exposed to acetaminophen. We observed 6.7 ± 0.8 waves at baseline in the acetaminophen-treated group (n = 13) and 5.9 ± 1.1 in the control group (n = 14) (*p* = 0.050).

**Fig. 2:**
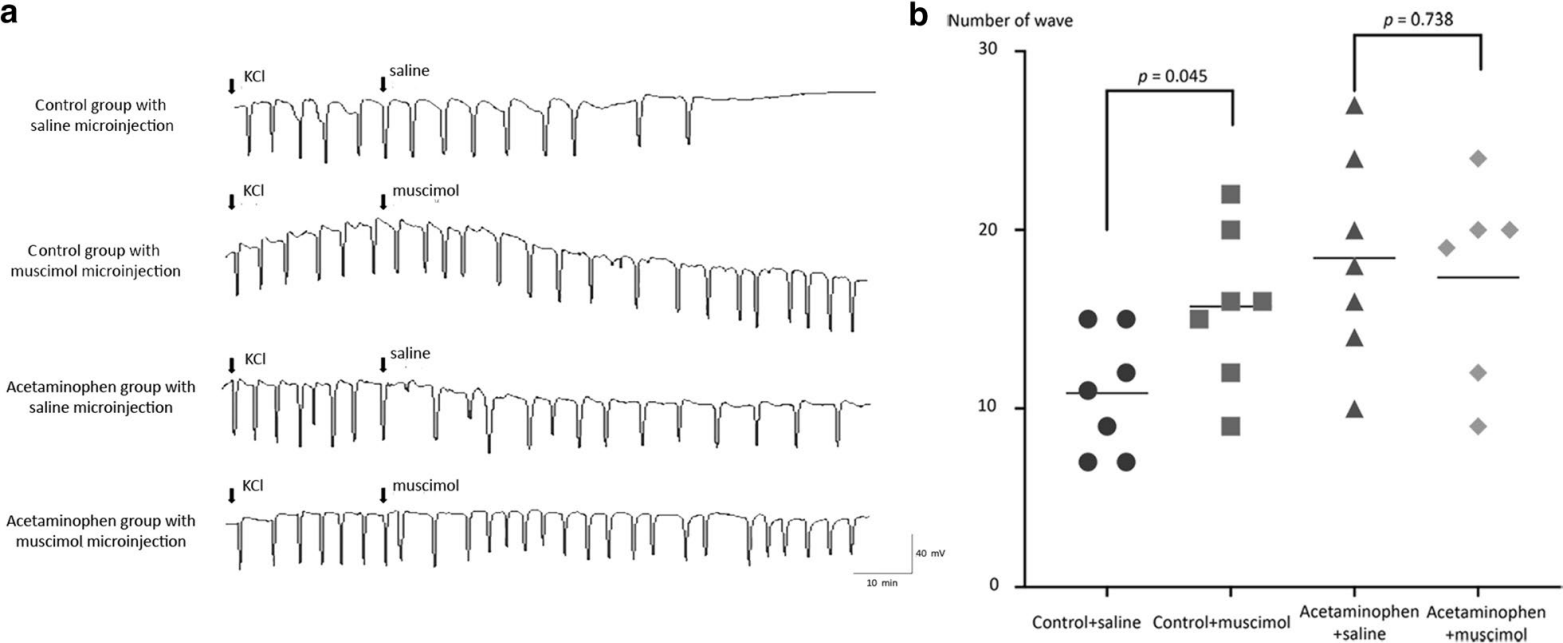
Effect of chronic treatment with acetaminophen on CSD development and modulating effect of muscimol microinjection into the NRM. Chronic treatment with acetaminophen significantly increased the number of cortical spreading depression (CSD) waves during the 30 min baseline period. In the saline-treated vehicle-control rats, the number of CSD waves within 90 min after the muscimol microinjection (15.7 ± 4.4; n = 7) was greater than that in the rats microinjected with saline vehicle control (10.9 ± 3.4; n = 7, *p* = 0.045). An enhancing effect of muscimol on CSD development was not observed in the acetaminophen-treated group. In the acetaminophen-treated group the number of CSD waves within 90 min after muscimol and saline microinjection was 17.3 ± 5.6 and 18.4 ± 5.8 respectively (*p* = 0.738). **a** Representative recordings comparing the effect of inhibiting the nucleus raphe magnus (NRM) with muscimol on CSD. **b** Scatterplots comparing the number of CSD waves after microinjection of muscimol or saline vehicle control into the NRM in the control group and the group of rats treated chronically with acetaminophen

Microinjection of muscimol into the NRM produced different effects in acetaminophen-treated and control rats. In the control rats, the number of CSD waves within 90 min after the microinjection of muscimol (15.7 ± 4.4; n = 7) was greater than that in the rats microinjected with saline vehicle control (10.9 ± 3.4; n = 7, *p* = 0.045). An enhancing effect of muscimol on CSD development was not observed in the acetaminophen-treated group in which the total CSD waves within 90 min of muscimol microinjection was 17.3 ± 5.6 (n = 6) and 18.4 ± 5.8 after saline (n = 7) (*p* = 0.738) (Fig. 2).

### Effect of inhibiting serotonergic systems in the NRM on CSD-evoked Fos-IR in the TNC

**Fig. 3:**
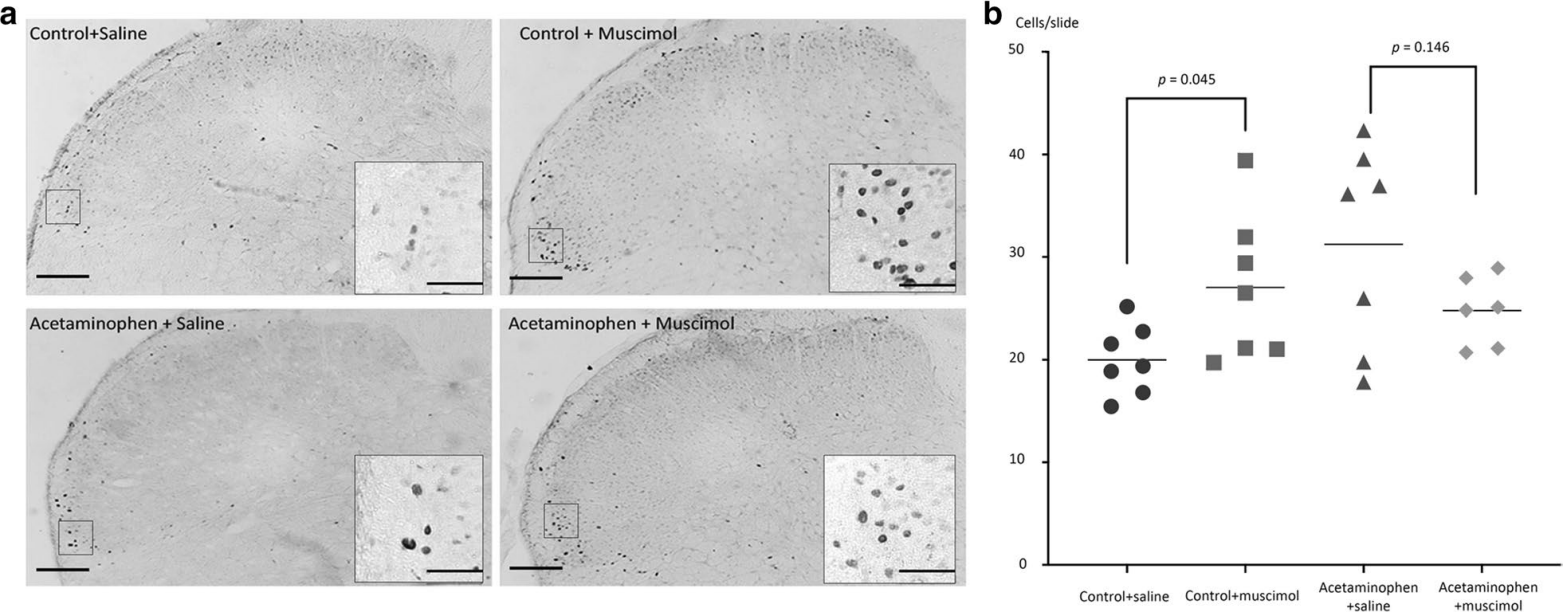
Effect of inhibiting the NRM on CSD-evoked Fos-IR in the TNC. Chronic treatment with acetaminophen increased the number of neurons with c-Fos-immunoreactivity (Fos-IR) in the ipsilateral trigeminal nucleus caudalis (TNC) evoked by cortical spreading depression (CSD) (31.2 ± 9.9 and 20.0 ± 3.4 cells/slide for acetaminophen-treated and saline-treated vehicle-control rats, respectively, *p* = 0.010). In the control rats, CSD evoked expression of Fos in the TNC was enhanced by muscimol microinjection into the nucleus raphe magnus (NRM) (27.0 ± 7.2 cells/slide, *p* = 0.045 compared with saline microinjected controls). By contrast, microinjection of muscimol into the NRM did not alter the number of CSD-evoked Fos expressing neurons in the acetaminophen-treated rats 24.8 ± 3.4 cells/slide (*p* = 0.146 compared with saline microinjected acetaminophen-treated rats). **a** Photomicrographs show patterns of Fos-IR in the four experimental groups (*scale bar* 100 µm in each section and 50 µm in the *inset*). **b** Scatterplots comparing the number of CSD-evoked Fos-IR cells per slide after microinjection of muscimol or saline vehicle control into the NRM of the control rats and the rats treated chronically with acetaminophen

Inhibiting of serotonergic systems in the NRM with muscimol significantly increased the number of Fos-IR cells in the ipsilateral TNC of saline-treated control rats (27.0 ± 7.2 cells/slide, *p* = 0.045 compared with saline microinjected controls; 20.0 ± 3.4 cells/slide). However, the enhancing effect of muscimol microinjection was not observed in the acetaminophen-treated rats where the number of Fos-IR cells was 24.8 ± 3.4 cells/slide (*p* = 0.146 compared with saline microinjected acetaminophen-treated rats; 31.2 ± 9.9 cells/slide) (Fig. 3). The number of Fos-IR cells in rats treated chronically with acetaminophen was significantly greater than that in saline microinjected control rats (*p* = 0.029) and the number of Fos-IR cells in rats treated chronically with acetaminophen and microinjected with muscimol was significantly greater than the number of Fos-IR cells in saline treated control rats microinjected with saline (*p* = 0.040).

### Effect of inhibiting serotonergic systems in the NRM on NTG-evoked neuronal firing in the TNC

**Fig. 4:**
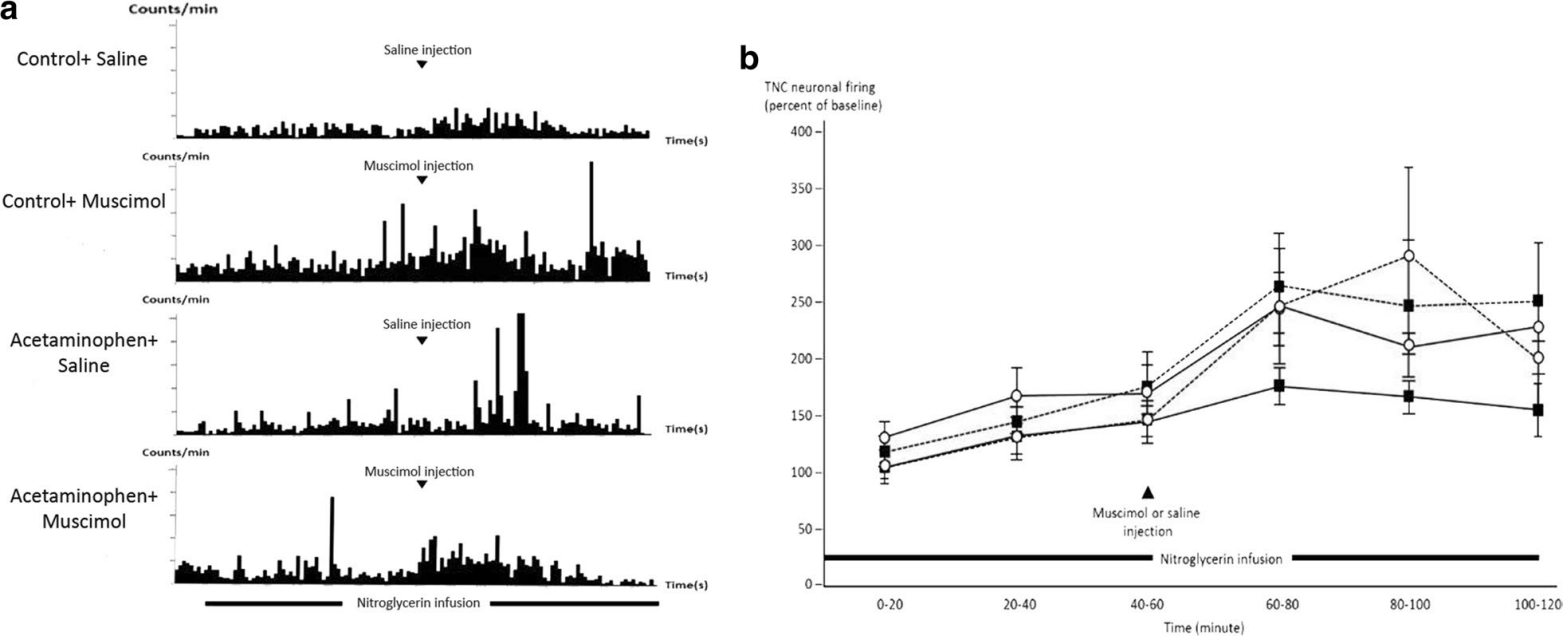
Effect of inhibiting the NRM on NTG-evoked TNC neuronal firing. **a** Representative recordings of neuronal firing in the trigeminal nucleus caudalis (TNC) evoked by infusing nitroglycerin (NTG) in four experimental groups. **b** Graph comparing the pattern of TNC neuronal firing evoked by infusing NTG in four experimental groups. In control rats, infusing NTG activated the firing of TNC neurons throughout the period of infusion (closed squares, solid line). The firing rate was increased in control rats with muscimol microinjection (closed squares, dash line; *p* = 0.002 compared with saline microinjection). Chronic treatment with acetaminophen increased the NTG-evoked neuronal firing in the TNC (open circles, solid line; *p* = 0.050, ANOVA for repeated measures). The effect of microinjection of muscimol into the NRM on increasing the TNC neuronal firing was not observed in rats that received chronic acetaminophen treatment (open circles, dashed line; *p* = 0.923 compared with saline microinjected acetaminophen-treated rats)

Intravenous systemic infusion with NTG increased the frequency of neuronal spikes in the TNC in both acetaminophen (n = 15) and saline control treated groups (n = 16). The activating effect occurred almost immediately after infusing NTG. NTG-induced neuronal firing in the TNC was significantly greater in rats with chronic acetaminophen exposure (*p* = 0.050, ANOVA for repeated measures) (Fig. 4).

Microinjection of muscimol into the NRM significantly increased neuronal firing in the TNC in the control groups (*p* = 0.002). Neuronal firing in the TNC in the 20 min postinjection was increased 182.1 ± 35.6% in the saline microinjection group (n = 8) and 268.2 ± 53.2% in the muscimol injection group (n = 8) compared with the average neuronal firing rate 20 min before microinjection (100%). The rates of neuronal firing in the TNC in the rats of the muscimol microinjected group were greater than those in rats of the saline microinjected group throughout the recording period. Microinjection of muscimol did not significantly alter rates of neuronal firing in the TNC in rats chronically treated with acetaminophen (*p* = 0.923). In acetaminophen-treated rats, neuronal firing in the TNC in the 20 min postinjection period was 253.7 ± 67.2% for rats microinjected with saline (n = 7) and 249.5 ± 96.4% in rats microinjected with muscimol (n = 8). At 40 min postinjection, the rate was 295.0 ± 157.7% in the muscimol-microinjected group, and higher than that in the saline-microinjected group (216.9 ± 18.5%) compared with the average neuronal firing rate 20 min before microinjection (100%), although the difference was not significant (*p* = 0.206) (Fig. 4).

### Effect of inhibiting serotonergic systems in the NRM on NTG-evoked Fos-IR in the TNC

**Fig. 5:**
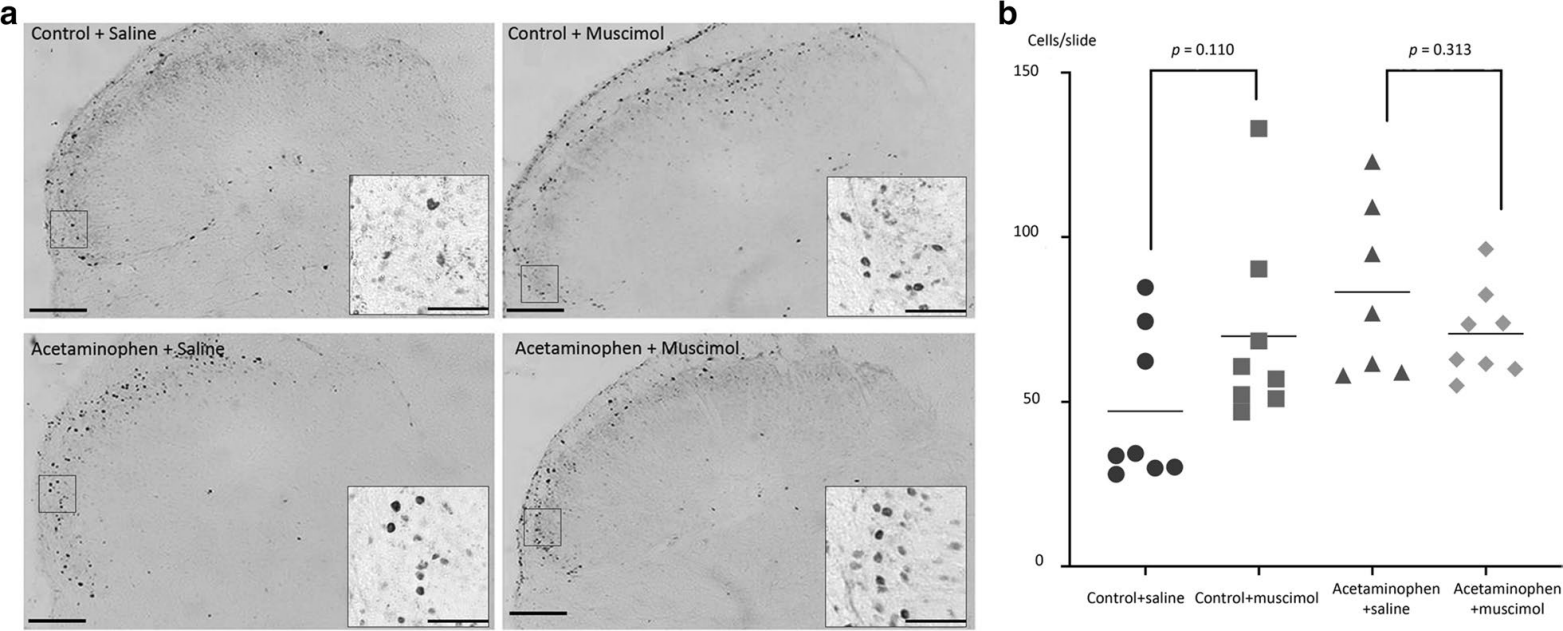
Effect of inhibiting the NRM on NTG-evoked Fos-IR in the TNC. **a** Photomicrograph shows patterns of c-Fos-immunoreactivity (Fos-IR) in four experimental groups (*scale bar* 100 µm in each section and 50 µm in the *inset*). **b** Scatterplots comparing the number of Fos-IR cells in four experimental groups. Chronic treatment with acetaminophen increased the number of nitroglycerin (NTG)-evoked Fos-IR neurons in the TNC. Microinjection of muscimol into the NRM did not alter the number of NTG-evoked Fos-IR neurons in the saline-treated vehicle-control and acetaminophen-treated rats

The number of Fos-IR cells in the acetaminophen-treated group (81.4 ± 28.6 cells/slide) was significantly greater than that of the control group (45.6 ± 24.8 cells/slide) (*p* = 0.019) (Fig. 5). Microinjection of muscimol into the NRM did not significantly change the number of NTG-evoked Fos expressing neurons in the saline-vehicle treated control (*p* = 0.110) and acetaminophen-treated groups (*p* = 0.313) (Fig. 5).

## Discussion

The present study demonstrated an involvement of the NRM on cortical excitability, as evident by an increase in CSD frequency, and excitability of nociceptive neurons, induced by initiating CSD or systemic infusion of NTG, in the TNC of rats chronically treated with acetaminophen. In saline vehicle-treated control rats, inhibiting serotonergic systems in the NRM with muscimol significantly increased CSD and NTG-induced neuronal firing in the TNC. These effects of microinjecting muscimol into the NRM were not observed in rats treated chronically with acetaminophen.

The results of the present study support our previous findings of the effect of chronic exposure to analgesics in increasing the excitability of neurons in the cerebral cortex and central trigeminal nociceptive pathway [3, 14]. Specifically, using our CSD model of migraine, we showed that chronic exposure to acetaminophen increased CSD development and the number of CSD-evoked Fos-IR neurons in the TNC. Based on this model, it is not possible to conclude whether the facilitation of trigeminal nociceptive pathway was caused by a direct effect upon the trigeminal nociceptive system or indirectly via the increased CSD development. To investigate this matter, we used an NTG infusion model of migraine in the present study to circumvent the effect of CSD activation. The increased neuronal firing and number of Fos-IR neurons in the TNC observed after infusion of NTG indicated that chronic exposure to analgesics such as acetaminophen might affect the trigeminal nociceptive system directly.

Here, we showed that the NRM has a powerful influence on cortical excitability and trigeminal nociceptive pathway. Neurons in the TNC were inhibited by direct microinjection of muscimol into the NRM. In saline-treated control rats, muscimol microinjection enhanced CSD development, increased NTG-evoked TNC neuronal firing, and Fos-IR in the TNC as evoked by CSD. These findings are consistent with those of previous studies, which showed that microinjection of a GABA_A_ receptor agonist into the NRM facilitated craniovascular nociceptive transmission [15]. This evidence confirms an important role of the NRM in modulating the sensitivity of cortical and trigeminal nociceptive neurons.

Our present findings also suggest that chronic exposure to analgesics may alter the function of the NRM. In the acetaminophen-treated rats, microinjection of muscimol into the NRM neither enhanced the development of CSD nor NTG-evoked trigeminal neuronal firing. The NRM is central to the serotoninergic system of the brainstem. This system has widespread termination including in the cortical, subcortical, and spinal areas. A substantial number of studies has revealed that the chronic use of a medication that is used for acute abortive treatment of migraine can affect the serotonergic system. Chronic administration of acetaminophen alters the amount of serotonin and its metabolites in selected brain areas [16]. Chronic exposure to acetaminophen upregulates pronociceptive 5HT_2A_ receptors in the cerebral cortex and trigeminal ganglion [14]. Chronic treatment with rizatriptan, a 5-HT_1B/1D_ receptor agonist decreases the amount of serotonin and upregulates 5-HT_2A_ receptors, which correlated positively with activation of Fos expression [17].

An imbalance in the function of the NRM reducing serotonergic output, may underlie the cortical hyperexcitation and facilitation of the trigeminal nociceptive system observed in rats exposed chronically to acetaminophen. Rats with decreased serotonin levels show increased development of CSD as a measure of cortical hyperexcitability and increased CSD-evoked Fos expression in the TNC [9]. Inhibiting NO production can reduce the development of CSD [18]. Reduced levels of serotonin may upregulate pronociceptive 5-HT_2A_ receptor expression in the cortex and trigeminal system. Upregulated expression of NOS and increased development of CSD may result from activation of this pronociceptive receptor [19]. Nociceptive traffic in the trigeminal system is also facilitated by low levels of serotonin. Rats with levels of serotonin depleted by inhibiting tryptophan hydroxylase increase the meningeal inflammation-evoked expression of Fos and phosphorylation of the NR1 NMDA-receptor subunit in neurons in the TNC [20]. Rats with depleted serotonin levels had increased CGRP expression in the trigeminal ganglion and CGRP release evoked by CSD [21, 22]. In human experiments, volunteers with acutely depleted tryptophan had a significantly decreased pain threshold and tolerance in response to heat from a thermode supporting a role for serotonin in pain modulation. There was a direct correlation between the reduction in plasma tryptophan levels and thermode temperature that caused pain [23].

## Conclusion

The present study suggests a role for the NRM in modulating the excitability of neurons in the cortex and TNC. Chronic exposure to acetaminophen compromises the function of this system. The medication-induced dysfunction of the NRM is a possible mechanism underlying the pathogenesis of MOH.

## Data Availability

The data supporting the results are presented in the text and figures, raw datasets used and analyzed during the current study are available from the corresponding author on reasonable request.

## Abbreviations

MOH: medication overuse headache
Fos-IR: c-Fos-immunoreactivity
TNC: trigeminal nucleus caudalis
NTG: nitroglycerin
CSD: cortical spreading depression
NRM: nucleus raphe magnus
SEP: somatosensory evoked potentials
5-HT: serotonin
NOS: nitric oxide synthase
CGRP: calcitonin gene-related peptide
DC: direct current

## References

[1] Headache Classification Committee of the International Headache Society (2018). The international classification of headache disorders, 3^rd^ ed. Cephalalgia.

[2] Kristoffersen ES, Lundqvist C (2014). Medication overuse headache: epidemiology, diagnosis and treatment. Ther Adv Drug Saf.

[3] Okada-Ogawa A, Porreca F, Meng ID (2009). Sustained morphine-induced sensitization and loss of diffuse noxious inhibitory controls in dura-sensitive medullary dorsal horn neurons. J Neurosci.

[4] De Felice M, Ossipov MH, Wang R, Lai J, Chichorro J, Meng I (2010). Triptan-induced latent sensitization: a possible basis for medication overuse headache. Ann Neurol.

[5] Supornsilpchai W, le Grand SM, Srikiatkhachorn A (2010). Cortical hyperexcitability and mechanism of medication-overuse headache. Cephalalgia.

[6] Vibulyaseck S, Bongsebandhu-phubhakdi S, le Grand SM, Srikiatkhachorn A (2014). Potential risk of dihydroergotamine causing medication overuse headache: preclinical evidence. Asian Biomed.

[7] Ayzenberg I, Obermann M, Nyhuis P, Gastpar M, Limmroth V, Diener HC (2006). Central sensitization of the trigeminal and somatic nociceptive systems in medication overuse headache mainly involves cerebral supraspinal structures. Cephalalgia.

[8] Coppola G, Currà A, Di Lorenzo C, Parisi V, Gorini M, Sava SL (2010). Abnormal cortical responses to somatosensory stimulation in medication-overuse headache. BMC Neurol.

[9] Supornsilpchai W, Sanguanrangsirikul S, Maneesri S, Srikiatkhachorn A (2006). Serotonin depletion, cortical spreading depression and trigeminal nociception. Headache.

[10] Saengjaroentham C, Supornsilpchai W, Ji-Au W, Srikiatkhachorn A, Maneesri-le Grand S (2015). Serotonin depletion can enhance the cerebrovascular responses induced by cortical spreading depression via the nitric oxide pathway. Int J Neurosci.

[11] D’Andrea G, D’Amico D, Bussone G, Bolner A, Aguggia M, Saracco MG (2014). Tryptamine levels are low in plasma of chronic migraine and chronic tension-type headache. Neurol Sci.

[12] Srikiatkhachorn A, Anthony M (1996). Platelet serotonin in patients with analgesic-induced headache. Cephalalgia.

[13] Srikiatkhachorn A, Anthony M (1996). Serotonin receptor adaptation in patients with analgesic-induced headache. Cephalalgia.

[14] Supornsilpchai W, le Grand MS, Srikiatkhachorn A (2010). Involvement of pro-nociceptive 5-HT_2A_ receptor in the pathogenesis of medication-overuse headache. Headache.

[15] Supronsinchai W, Storer RJ, Hoffmann J, Andreou A, Akerman S, Goadsby P (2013). GABA_A_ receptors in the nucleus raphe magnus modulate firing of neurons in the trigeminocervical complex. J Headache Pain.

[16] Blecharz-Klin K, Piechal A, Pyrzanowska J, Joniec-Maciejak I, Kiliszek P, Widy-Tyszkiewicz E (2013). Paracetamol—the outcome on neurotransmission and spatial learning in rats. Behav Brain Res.

[17] Su M, Ran Y, Han X, Liu Y, Zhang X, Tan Q (2016). Rizatriptan overuse promotes hyperalgesia induced by dural inflammatory stimulation in rats by modulation of the serotonin system. Eur J Neurosci.

[18] Le Grand SM, Supornsilpchai W, Saengjaroentham C, Srikiatkhachorn A (2011). Serotonin depletion leads to cortical hyperexcitability and trigeminal nociceptive facilitation via the nitric oxide pathway. Headache.

[19] Srikiatkhachorn A, Suwattanasophon C, Ruangpattanatawee A, Phansuwan-Pujito P (2002). 5-HT_2A_ receptor activation and nitric oxide synthesis: a possible mechanism determining migraine attacks. Headache.

[20] Maneepak M, le Grand SM, Srikiatkhachorn A (2009). Serotonin depletion increases nociception-evoked trigeminal NMDA receptor phosphorylation. Headache.

[21] Le Grand SM, Saengjaroentham C, Supronsilpchai W, Srikiatkhachorn A (2010). The increase in the calcitonin gene-related peptide Immunoreactivity following the CSD activation in the serotonin depleted state. J Headache Pain.

[22] Saengjaroentham C, Supornsilpchai W, Srikiatkhachorn A, le Grand SM (2012). The effect of the serotonin depletion on the cortical spreading depression induced the release of calcitonin gene related peptide (CGRP) and the ultrastructural alteration of the cerebral microvessels. J Neurochem.

[23] Martin SL, Power A, Boyle Y, Anderson IM, Silverdale MA, Jones AKP (2017). 5-HT modulation of pain perception in humans. Psychopharmacology.

[24] Wanasuntronwong A, Jansri U, Srikiatkhachorn A (2017). Neural hyperactivity in the amygdala induced by chronic treatment of rats with analgesics may elucidate the mechanisms underlying psychiatric comorbidities associated with medication-overuse headache. BMC Neurosci.

[25] Paxinos G, Watson C (1986). The rat brain in stereotaxic coordinates.

